# Prognostic alternative mRNA splicing in lung adenocarcinoma

**DOI:** 10.3389/fonc.2025.1579017

**Published:** 2025-11-18

**Authors:** Panke Su, Pei Xu, Deying Xu

**Affiliations:** Department of Clinical Laboratory, The First Affiliated Hospital, and College of Clinical Medicine of Henan University of Science and Technology, Luoyang, China

**Keywords:** alternative splicing, lung adenocarcinoma, prognosis, risk model, therapy

## Abstract

**Background:**

Alternative splicing (AS) of mRNA has emerged as a promising biomarker for various tumors, playing a crucial role throughout nearly all stages of tumor progression and influencing the tumor immune microenvironment (TIME). Our study was designed to develop an AS-based signature for accurate prognosis prediction in lung adenocarcinoma (LUAD) patients, to delineate the associated immune cell landscape, and to pinpoint promising drug targets.

**Methods:**

Prognostic alternative splicing events (PASEs) were identified through univariate Cox regression analysis of RNA-Seq data from The Cancer Genome Atlas (TCGA). These PASEs were incorporated into a least absolute shrinkage and selection operator–Cox proportional hazards model to develop a prognostic signature. Experimental validation was performed using reverse transcription quantitative polymerase chain reaction, immunohistochemistry, and functional assays *in vitro* and *in vivo*.

**Results:**

A total of 13 PASEs were selected to form the prognostic signature, which demonstrated excellent predictive power for 1-, 2-, and 3-year overall survival (OS), with area under the receiver operating characteristic curve values of 0.776, 0.751, and 0.767, respectively. High-risk patients, identified by the signature, showed significantly decreased stromal, immune, and combined scores; increased tumor purity (*P*< 0.001); a reduced prevalence of various immune cell types; diminished immune cell activity; and decreased expression of immune checkpoint genes. Notably, elevated expression of cyclin-dependent kinase inhibitor 2A (CDKN2A), a gene associated with PASEs, correlated with poorer OS and significantly higher infiltration of CD8^+^ T cells, activated memory CD4^+^ T cells, and M1 macrophages compared to patients with lower expression. Further validation studies confirmed increased CDKN2A levels in LUAD tissues, with CDKN2A protein expression inversely correlated with LUAD prognosis (hazard ratio = 2.737; 95% confidence interval, 1.524–4.915; *P* = 0.0002). CDKN2A was found to promote LUAD progression *in vitro*. Molecular docking identified YM-201636 and VE-822 (Berzosertib) as potential drugs targeting CDKN2A, both showing promise for LUAD treatment *in vivo*.

**Conclusion:**

PASEs constitute a comprehensive biomarker for predicting prognosis and monitoring the TIME in LUAD patients. Specifically, CDKN2A stands out as a potential prognostic biomarker and drug target for LUAD.

## 1 Introduction

Lung carcinoma is the most common tumor in the world with high mortality (1, 2) and is the leading cause of death for men and the second leading cause of death for women following breast cancer (3, 4). Lung carcinoma can be categorized into small cell lung carcinoma (SCLC) and non–small cell lung carcinoma (NSCLC), of which NSCLC is the most prevalent type, accounting for about 85% of lung carcinomas (5). Advances in precision medicine allow gene-based classification of cancer subtypes. NSCLC is considered a highly heterogeneous disease as diverse phenotypes and genotypes are present in patients with each NSCLC subtype, including the two most common histological types, lung adenocarcinoma (LUAD) and lung squamous cell carcinoma (6). Compared to other NSCLC subtypes, LUAD has been reported to have close associations with genomic variations, including *TP53*, *KRAS*, *EGFR*, *NF1*, *BRAF*, *MET*, and *RIT* mutations (7) that frequently occur and those rarely reported, such as *HOXA4* and *MST1* mutations (8). However, lung carcinoma mortality remains high even after the applications of many new molecular targeted therapies and immunotherapy agents. Early detection of potentially tumorigenic genomic or genetic changes using new prognostic markers may maximize the efficacy of personalized treatment for longer survival of lung carcinoma patients.

Alternative splicing (AS) is a mechanism by which eukaryotic mRNA isoforms are generated from a single gene by removal of introns and selective joining of specific exons (9), thus controlling gene expressions (10) via disturbing mRNA stability, localization, and translation (11). It is also a post-transcriptional process generating multiple protein products from a single gene encoding, resulting in protein diversity (12). Eukaryotic cells utilize protein diversity to support the functional complexity of genes (13). Physiologically, more than 95% of human genes are associated with AS events. However, tumor cells use abnormal AS events for tumor progression to disrupt metabolism and cell cycle control, stimulate invasion and metastasis of cancer cells while inhibiting apoptosis, and promote angiogenesis, thus reconstructing the tumor microenvironment (14). These events have been shown to reduce the efficacy of targeted therapy, chemotherapy, and hormone therapy or immunotherapy (15).

Growing evidence shows that mutations in AS events have the potential to become neoepitopes for immunotherapy (16). Type- or subtype-specific genes undergoing AS events in cancer cells often encode components of core splicing machinery or splicing factors (SFs) with regulatory sites and result in accumulation of mutations that lead to cancer (17), thus having great prognostic significance. AS events are also significantly associated with immune microenvironment formation, the infiltration of immune cells, and their lytic activity on tumor cells (18). Cancer-specific AS has the potential to predict the efficacy of anticancer therapies (19). These findings suggest that cancer-specific AS events also have prognostic potential.

For far so long, there have been a few published reports that comprehensively analyze AS events in LUAD and their clinical significance. Most studies used The Cancer Genome Atlas (TCGA) database and explored the impact of AS events on other tumor types rather than identifying an efficient biomarker and drug targets (20, 21). A recent study ascertained AS as a potential prognostic marker of LUAD (21). In this study, our objective was to develop a risk model utilizing transcriptome and clinical data from TCGA, specifically focusing on prognostic AS events in LUAD. Additionally, we aimed to validate the model’s efficacy in predicting survival and characterizing the immune cell landscape. Notably, we conducted further validation of the prognostic significance and functional role of the identified CDKN2A molecule and explored its potential as a therapeutic target in LUAD treatment *in vivo*.

## 2 Materials and methods

### 2.1 Data collection

We collected RNA-Seq and clinical data of 535 tumor tissues and 59 para-carcinoma tissues of LUAD patients from TCGA-GDC (portal.gdc.cancer.gov/projects/TCGA-LUAD; Project ID: TCGA-LUAD). AS data were downloaded from TCGA SpliceSeq (https://bioinformatics.mdanderson.org/TCGASpliceSeq/), from which percent spliced-in (PSI) values for splice events on samples were obtained. Samples with a PSI value of ≥ 75% were included in further analysis, including 513 tumorous and 59 tumor-adjacent normal tissues.

### 2.2 Generation of a prognostic model

Cases without information on survival time or states or AS data were excluded from our analysis. AS and survival data (*n* = 513) were integrated and subjected to univariate Cox regression to screen out prognostic alternative splicing events (PASEs; a *P*-value threshold of 0.05 for screening), which were shown in UpSet, volcano, and bubble plots. Least absolute shrinkage and selection operator (LASSO) regression analysis was utilized to generate a PASE-based risk model and prevent overfitting and improve the accuracy of this model. The best *λ* value was obtained for the risk model using 10-fold cross-validation in the “glmnet” package. Based on multivariate Cox regression analysis, the risk score of each patient was calculated using the following formula: riskScore = $\sum_{i}^{\text{n}}PSI*\beta i$ where *n*, PSI, and *βi* represent the number of AS events, the PSI value, and the regression coefficient, respectively. Then, the median riskScore was calculated. Patients with a riskScore greater than the median value were assigned to the high-risk group; otherwise, they were classified into the low-risk group.

### 2.3 Validation of the risk model

We assessed the accuracy of the risk model in survival prediction using Kaplan–Meier survival curves drawn by the survival package in R software and receiver operating characteristic (ROC) curves. Risk curves, risk heatmaps, and survival plots of LUAD patients were plotted according to the ranking of patients’ riskScores. The independence of the risk model in survival prediction was validated using univariate and multivariate Cox regression.

Kaplan–Meier survival curves were plotted to obtain ROC curves. The accuracy of the risk model and other clinicopathological factors (age, gender, and tumor stage) in 1-, 2-, and 3-year survival prediction was determined by the area under the ROC curve (AUC) values. The correlation analysis of patients’ riskScore with clinical traits was performed to validate whether the risk model can discriminate clinical traits associated with increased LUAD risk. We constructed a nomogram based on the sum of the scores of all clinical traits included. Its efficacy in survival prediction was also evaluated.

### 2.4 Validation of the risk model in immune cell landscape characterization

Stromal, immune, and summed scores for tumor purity were compared between high- and low-risk groups in the estimate package, and the results were shown in violin box plots. We calculated the relative proportion of immune cell types of each tumor sample in CIBERSORT (22). Single-sample gene set enrichment analysis (ssGSEA) was utilized to obtain the immune score of each patient. A higher score represented an increased number of immune cells or enhanced immune-related functions. Differences in immune cell subpopulations, immune activity, and immune checkpoint gene expressions were compared between the high- and low-risk groups.

### 2.5 Identification of PASE-related genes

We also identified differentially expressed genes (DEGs) associated with the key PASEs included in the risk model and assessed their correlations with survival risk and immune cell infiltration. PASE-related DEGs in LUAD were selected with Limma in R. *P*-values of genes were corrected by the Benjamini-Hochberg method. Genes with |logFC| > 1 and *P*< 0.05 were selected. The correlation analyses of PASE-related DEGs with patient survival, immune cell subpopulations, and immune score were performed. TIMER (http://timer.comp-genomics.org) algorithm was employed to analyze the correlations of differential genes with immune checkpoint gene expressions (23). Cytoscape visualized the correlation analysis between PASEs significantly associated with LUAD and expressions of SFs. SFs with a correlation coefficient |R| > 0.6 and *P*< 0.001 were selected.

### 2.6 RT-qPCR

Validation studies were carried out on one LUAD tissue chip (Cat. No. cDNA-HLugA030PG01, Shanghai Outdo Biotech, Shanghai, China; each tissue chip has 15 LUAD tissue points together with 15 adjacent normal tissue points). The primer sequences were *CDKN2A* forward: 5′-GGGTTTTCGTGGTTCACATCC-3′ and reverse: 5′-CTAGACGCTGGCTCCTCAGTA-3′, product length of 105 bp; and *GAPDH* forward: CTGGGCTACACTGAGCACC and reverse: AAGTGGTCGTTGAGGGCAATG, product length of 101 bp. The 25-μL total PCR reaction system contains 9 μL of RNase-free H_2_O, 0.5 μL of upstream/downstream primers (10 mM), 2.5 μL cDNA template, and 12.5 μL of SYBR Green Master (ROX) (Roche, Switzerland), and the reaction conditions were as follows: 10 min of pre-denaturation at 95°C, 15 s of denaturation at 95°C, and 1 min of reaction at 60°C for a total of 30 cycles. The 2^−ΔΔCt^ method served for calculating the relative gene expression.

### 2.7 IHC

The protein expression analysis was conducted under the assistance of high-throughput LUAD tissue microarray (Cat. No. HLugA180Su05, comprising 94 LUAD and 86 adjacent tissue spots; Shanghai Outdo Biotech, Shanghai, China). For the clinical information of LUAD tissue microarray, the operation period spanned from July 2004 to June 2009, with the follow-up conducted in August 2014; the duration of follow-up ranged from 5 to 10 years. Participants have not been treated by chemotherapy, radiotherapy, or others prior to surgery. The EnVision DAB test kit (MXB Biotechnologies, Fuzhou, China) served for the immunohistochemical analysis on 4-μm paraffin-embedded tissues/cells fixed in formalin, taking phosphate-buffered saline (PBS) as a negative control. Rabbit monoclonal to CDKN2A (EPR24167-43, Abcam) Immunoglobulin G (IgG) was diluted to 1:200. A light microscope (Olympus IX73, Japan) served for the photographing. Two senior pathologists took charge of scoring cells in these sections considering the coloring status and color development degree, and the scoring criteria were based on the published study (24).

### 2.8 Cell lines, cell culture, and lentiviral vector infection

Human LUAD cells (A549) were obtained from the American Tissue Culture Collection and validated through short tandem repeat (STR) profiling. The A549 cells were cultured in complete RPMI-1640 growth media (ThermoFisher, USA) supplemented with 10% heat-inactivated fetal bovine serum (FBS; Sigma-Aldrich, NZ). Additionally, the media contained 2 mM L-glutamine and 1% streptomycin/penicillin (100 μg/mL, Sigma-Aldrich, AU). The cells were maintained in a humidified incubator at 5% CO_2_, 95% O_2_, and a steady temperature of 37°C. CDKN2A overexpression and interfering lentiviral vectors were all provided by GENECHEM (Shanghai, China). Lentivirus infection followed the instructions provided by GENECHEM.

### 2.9 CCK-8

Log-phase cells were seeded in a 96-well plate at a density of 5 × 10^3^ cells/well, with a volume of 100 μL per well. Experimental groups included overexpression groups (CDKN2A-OE group, Mock group) and short hairpin RNA (shRNA) groups (Si-CDKN2A group, Si-NC group), with five replicate wells per group. After culturing for 0, 24, 48, 72, and 96 h, 10 μL of Cell Counting Kit-8 (CCK-8) reagent (Beyotime Biotechnology, Shanghai, China) was added to each well and incubated for an additional 3 h. An Enzyme-Linked Immunosorbent Assay (ELISA) reader (Thermo Scientific) measured the optical density (OD) at 450-nm and 630-nm wavelengths. The experiment was repeated three times, and cell growth curves were plotted with OD values on the y-axis and time on the x-axis.

### 2.10 Scratch assay

Log-phase A549 cells were plated in a six-well plate. A 10-μL pipette tip was employed to scratch the surface of A549 cell groups, followed by continued incubation for 48 h. The scratch widths were observed under a microscope (Olympus IX73, Japan) at 0 and 48 h. An incomplete medium-treated group served as the control to compare healing rates among different groups. Five randomly selected high-magnification fields of view were counted to ascertain the average width.

### 2.11 Transwell assay

Take 300 μL of serum-free medium, add 60 μL of Matrigel, mix well at 4°C, and add 100 μL to each upper chamber (three chambers in total) and then incubate at 37°C for 4–5 h. Digest breast cancer cells, wash three times with serum-free medium, count, and prepare a cell suspension. Then, wash the Matrigel (added for the invasion assay) (Corning, New York, USA) once with serum-free medium, and add 100 μL of the cell suspension to each well. Subsequently, add 500 μL of medium containing 20% FBS to the lower chamber. Incubate in a 37°C incubator for 20 to 24 h. Remove the transwell chamber, wash twice with PBS, and fix with 5% glutaraldehyde at 4°C. Stain with 0.1% crystal violet or Giemsa for 5 to 10 min at room temperature, wash twice with PBS, wipe off the cells from the upper surface with a cotton ball, and count in nine random fields under a microscope, summarize the results.

### 2.12 Molecular docking

The full-length amino acid sequence of protein CDKN2A was obtained from the UniProt database. This sequence was input into AlphaFold 3, where the optimal protein model was selected for analysis. The two-dimensional structure of CDKN2A was analyzed using PDBsum. Molecular docking simulations were performed to model the interactions between the wild-type structure of protein CDKN2A and small-molecule substrates using molecular docking software on a computer. Small-molecule structures were downloaded from the Selleckchem L1100-Inhibitor-Library and imported into a small-molecule database created in MOE v2022.02 software, where they underwent energy minimization. The three-dimensional structure of CDKN2A predicted by AlphaFold was imported into MOE software for pre-docking processing and optimization of the protein side chains. Docking was performed using the Dock module in MOE v2022.02.

### 2.13 Molecular dynamics

Molecular dynamics (MD) simulations lasting 100 ns were conducted using GROMACS 2020.6 software to further validate the docking results’ reliability and reasonableness. Parameters and topology files for the protein and small-molecule ligands were generated using Amber03 and Amber GAFF force fields, respectively. Periodic boundary conditions were set, and the protein was centered in a cubic box with a minimum distance of 1.0 nm from the edges, which was filled with water molecules at a density of 1. To achieve electrical neutrality, some water molecules were replaced with Na+ and Cl- ions at a concentration of 0.15 mol/L. The system’s energy was minimized using the steepest descent method to reduce any unreasonable contacts or atomic overlaps. Solvents and ions around the protein were pre-equilibrated in two stages: the first stage involved an NVT ensemble at 300 K and 100 ps to stabilize the temperature, followed by an NPT ensemble at 1 bar and 100 ps to stabilize the pressure. The leapfrog algorithm was used for integrative dynamics during MD simulations, conducted under isothermal and isobaric conditions (300 K and 1 bar) over 100 ns. Post-simulation, trajectories were centered on the protein and analyzed for root mean square deviation (RMSD). The interaction energy between the protein and the small-molecule complex was calculated using the gmx_MMPBSA v1.6.1 tool employing the single-trajectory (ST) method. Finally, the simulation trajectories were analyzed to compute contributions from van der Waals energy (VDWAALS), electrostatic energy (EPB), polarization energy (ENPOLAR), gas-phase energy (GGAS), and solvation energy (GSOLV).

### 2.14 Evaluation of antitumor effects *in vivo*

Female NOD.Cg-Prkdcscid Il2rgem1cya/Cya (NKG) mice (weighing 16 to 23 g, aged 6 weeks, n = 6) were sourced from Cyagen Biosciences (Suzhou, China). All *in vivo* experiments were conducted in strict adherence to the guidelines outlined in the National Institutes of Health Guide for the Care and Use of Laboratory Animals. The mice were provided with humane care, and the study protocol was approved by the Institutional Animal Care and Use Committee of Cyagen Biosciences. Orthotopic mouse models of LUAD were established using CDKN2A-OE luciferase-labeled human A549 cells administered via tail vein injection (0.5 M/100 μL). Twenty-eight days post-inoculation, the mice were randomly assigned to the YM201636 and VE-822 groups. The mice in the YM201636 and VE-822 groups received dosages of 2 mg/kg and 25 mg/kg, respectively, via tail vein injection once every other day for 14 days (total of seven injections). Subsequently, the mice were imaged using an *in vivo* imaging system to observe and measure the fluorescence signal.

### 2.15 Statistical analysis

All statistical analyses were performed in R-4.0.2 software (https://www.r-project.org/) and GraphPad Prism 10.2.3. The Kaplan–Meier method was utilized to calculate the overall survival (OS), and the logarithmic rank test was used to compare survival curves. An AUC value of > 0.60 was considered acceptable and that of > 0.70 was regarded to show significant effects (25, 26). Clinical characteristics were compared between the high- and low-risk groups. The *χ2* test, the logarithmic rank test, and a Cox proportional hazards regression model were employed to compare dichotomous variables. The significance level was set at *P*< 0.05.

## 3 Results

### 3.1 AS Events in LUAD

**Figure 1:**
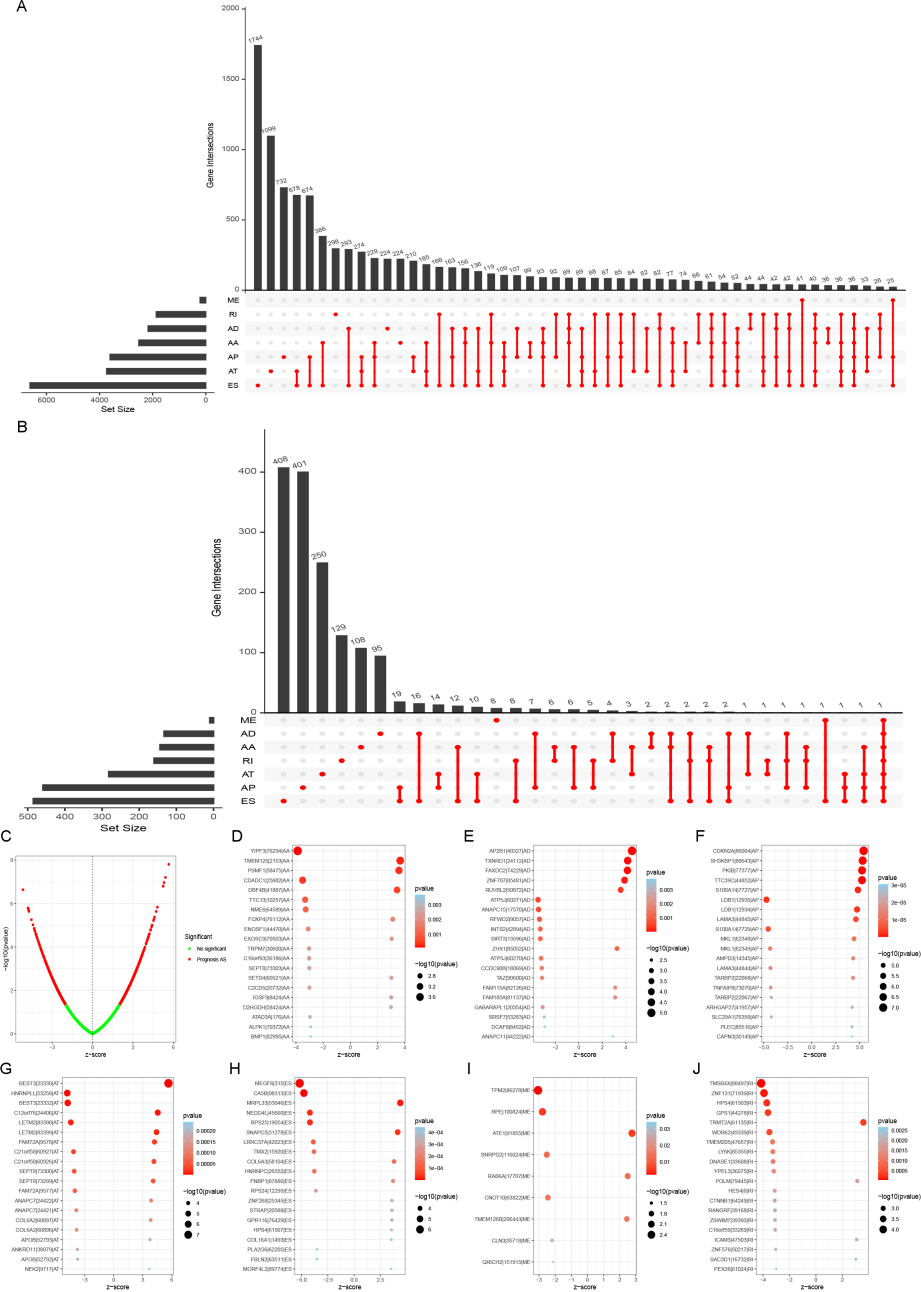
Visualization of alternative splicing (AS) in LUAD. **(A)** An UpSet plot depicting AS with PSI ≥ 75% in LUAD patients. **(B)** Through UpSet plot analysis, 1,666 AS events were identified as prognostic alternative splicing events (PASEs) using univariate Cox regression (*P*-value threshold< 0.05), with exon skipping (ES), alternative termination (AT), and alternative promoter (AP) representing the three most predominant PASE types. **(C)** Panel C presents all alternative splicing (AS) events in a volcano plot format, with red dots indicating statistically significant PASEs. **(D–J)** Bubble maps were employed to visualize highly significant PASEs across the seven categories, with the x-axis indicating the z-score and the y-axis denoting the genealternative splicing IDsplicing type. Bubble size reflects the −log10 (*P*-value) derived from univariate Cox regression analysis, whereas color intensity represents prognostic association: darker red hues indicate a higher likelihood of the AS (alternative splicing) event being linked to prognosis.

LUAD-associated AS events could be categorized into seven types, including alternate acceptor site (AA), alternate donor site (AD), alternate promoter (AP), alternate terminator (AT), exon skip (ES), mutually exclusive exons (ME), and retained intron (RI). A total of 19,523 AS events of 513 LUAD patients were identified and shown in the UpSet plot (**Figure 1A**). ES, AT, and AP events revealed the highest frequency among all AS types. Inside, 1,666 ASs were identified by univariate Cox regression (a *P*-value threshold of< 0.05) as PASEs, with ES, AT, and AP as the top three PASE types (**Figure 1B**). All AS events were visualized in a volcano plot, with red dots indicating statistically significant PASEs (**Figure 1C**). Univariate Cox regression analysis (threshold: *P*< 0.05) was performed to identify significant PASEs across seven types, which were then visualized in a bubble plot: the x-axis represents the z-score, whereas the y-axis displays the gene name | splicing event ID | splicing type (**Figures 1D–J**).

### 3.2 Generation and validation of a PASE-based risk model

**Figure 2:**
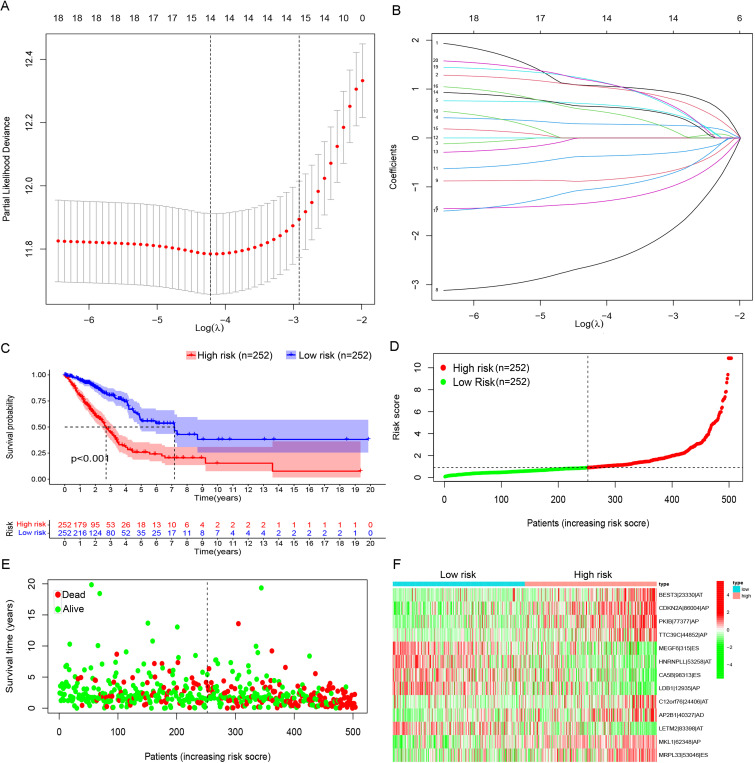
Prognostic model based on PASEs. **(A)** The Lambda value distribution plot of the 14 candidate PASEs demonstrated a bell-shaped curve with a central peak and symmetrical bilateral tapering, resembling a normal distribution. This pattern suggests a stable data generation process operating within expected variability limits. **(B)** The coefficient of PASEs were derived through LASSO regression analysis. **(C)** The Kaplan–Meier survival curves were stratified by risk groups (high vs. low) on the basis of the prognostic model, demonstrating significant divergence in clinical outcomes (log-rank *P*< 0.001). **(D)** Scatter plots illustrate the distribution of low-to-high risk values among LUAD patients. **(E)** Scatter plot displaying survival time and status in LUAD patients. **(F)** A heatmap shows the PSI of PASE in the LUAD prognostic model.

After integration of AS and survival data of LUAD patients, 14 candidate PASEs were selected from 1,666 PASEs using LASSO regression analysis (a *P*-value threshold of 0.05 for screening, **Figures 2A, B**). The 14 PASEs were subsequently included in multivariate Cox proportional hazards regression, and 13 PASEs were ultimately identified, including BEST3|23,330|AT, CDKN2A|86,004|AP, PKIB|77,377|AP, TTC39C|44,852|AP, MEGF6|315|ES, HNRNPLL|53,258|AT, CA5B|98,313|ES, LDB1|12,935|AP, C12orf76|24,406|AT, AP2B1|40,327|AD, LETM2|83,398|AT, MKL1|62,348|AP, and RPL33|53,046|ES. A 13-PASE–based signature was established, and the median riskScore of LUAD patients was 0.907.

The cohort of 513 LUAD patients was dichotomized into high- and low-risk groups using the median risk score (riskScore) as the optimal cutoff. This classification yielded balanced subgroups, with 252 patients in each risk category (high-risk: riskScore > median; low-risk: riskScore ≤ median). The median cutoff approach was selected to (1) maintain equal group sizes for statistical power, (2) minimize classification bias, and (3) align with established prognostic modeling conventions. Kaplan–Meier survival curves revealed that high-risk patients had a markedly lower OS rate than low-risk patients (**Figure 2C**). In the high-risk group, patients showed shorter OS time (**Figure 2D**) and a rising number of fatalities (**Figure 2E**) with increasing riskScore. Further, 8 of the 13 PASEs (BEST3|23,330|AT, CDKN2A|86,004|AP, PKIB|77,377|AP, TTC39C|44,852|AP, C12orf76|24,406|AT, AP2B1|40,327|AD, MKL1|62,348|AP, and RPL33|53,046|ES) showed increasing frequency with increasing riskScore, and the other five events (MEGF6|315|ES, HNRNPLL|53,258|AT, CA5B|98,313|ES, LDB1|12,935|AP, and LETM2|83,398|AT) exhibited the opposite trend (**Figure 2F**).

**Figure 3:**
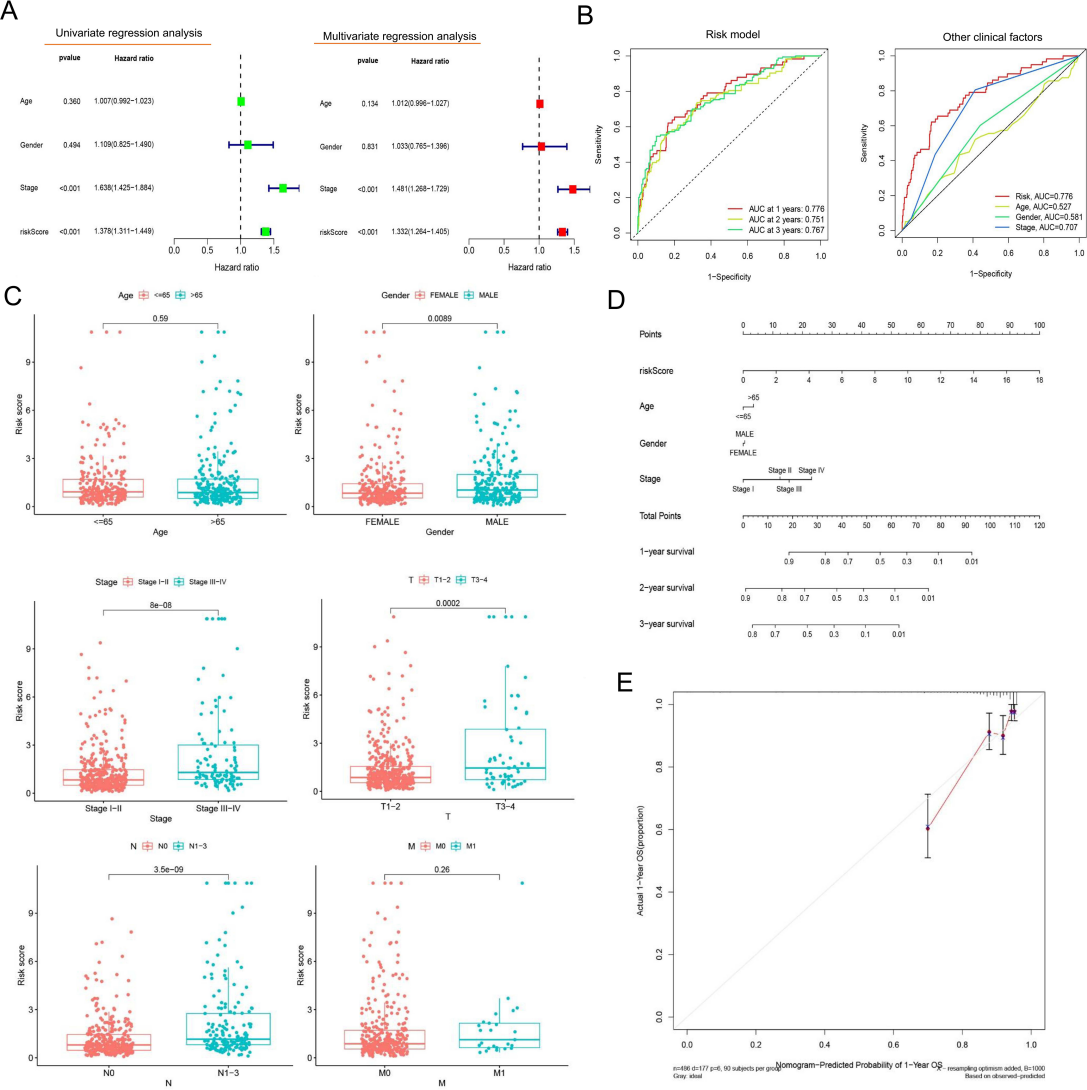
Evaluation of the prognostic risk model’s value. **(A)** Forest plots presenting the results of univariate and multivariate Cox regression analysis. The forest plots illustrate hazard ratios (HRs) with 95% confidence intervals (CIs) for each variable assessed in the prognostic model. **(B)** ROC curves comparing the prognostic model and other clinical factors. The area-under-the-ROC-curve (AUC) values quantify predictive accuracy, with the risk model outperforming traditional clinical factors at 1-, 3-, and 5-year survival intervals. **(C)** Comparative analysis of clinical characteristics between the high- and low-risk groups. Boxplots illustrate the distribution of age, whereas stacked bar charts depict categorical variables (gender, tumor stage, and TNM [tumor, node, metastasis] classification) stratified by the prognostic risk model’s stratification (high- vs. low-risk groups). **(D)** A line graph depicting survival probability over time. **(E)** The calibration curve. This plot assesses agreement between predicted and observed survival rates at a defined timepoint. Ideal calibration (45° dashed line) is compared to our model’s predictions (solid line), with minor deviations indicating high reliability.

Univariate and multivariate Cox regression confirmed that the risk model could predict the OS independent of age, gender, and tumor stage (*P*< 0.001, **Figure 3A**). The AUCs of the risk model in 1-, 2-, and 3-year OS prediction were 0.776, 0.751, and 0.767, indicating satisfactory accuracy in prognostic prediction, higher than those of other clinical factors (**Figure 3B**). In subgroup comparisons, male patients had a significantly higher riskScore than female patients; an increased riskScore could also be observed in stage III–IV vs. I–II patients, stage T3–T4 vs. T1–T2 patients, and patients with metastatic lymph nodes vs. non-metastatic LUAD patients (**Figure 3C**). Thus, the riskScore was closely associated with gender, tumor stage and size, and lymph node metastasis (**Figure 3C**). The clinical nomogram based on the three clinical factors also showed satisfactory efficacy in 1-, 2-, and 3-year OS prediction (**Figure 3D**), as further supported by the calibration curves (**Figure 3E**).

### 3.3 Relationship between TIME and risk model

**Figure 4:**
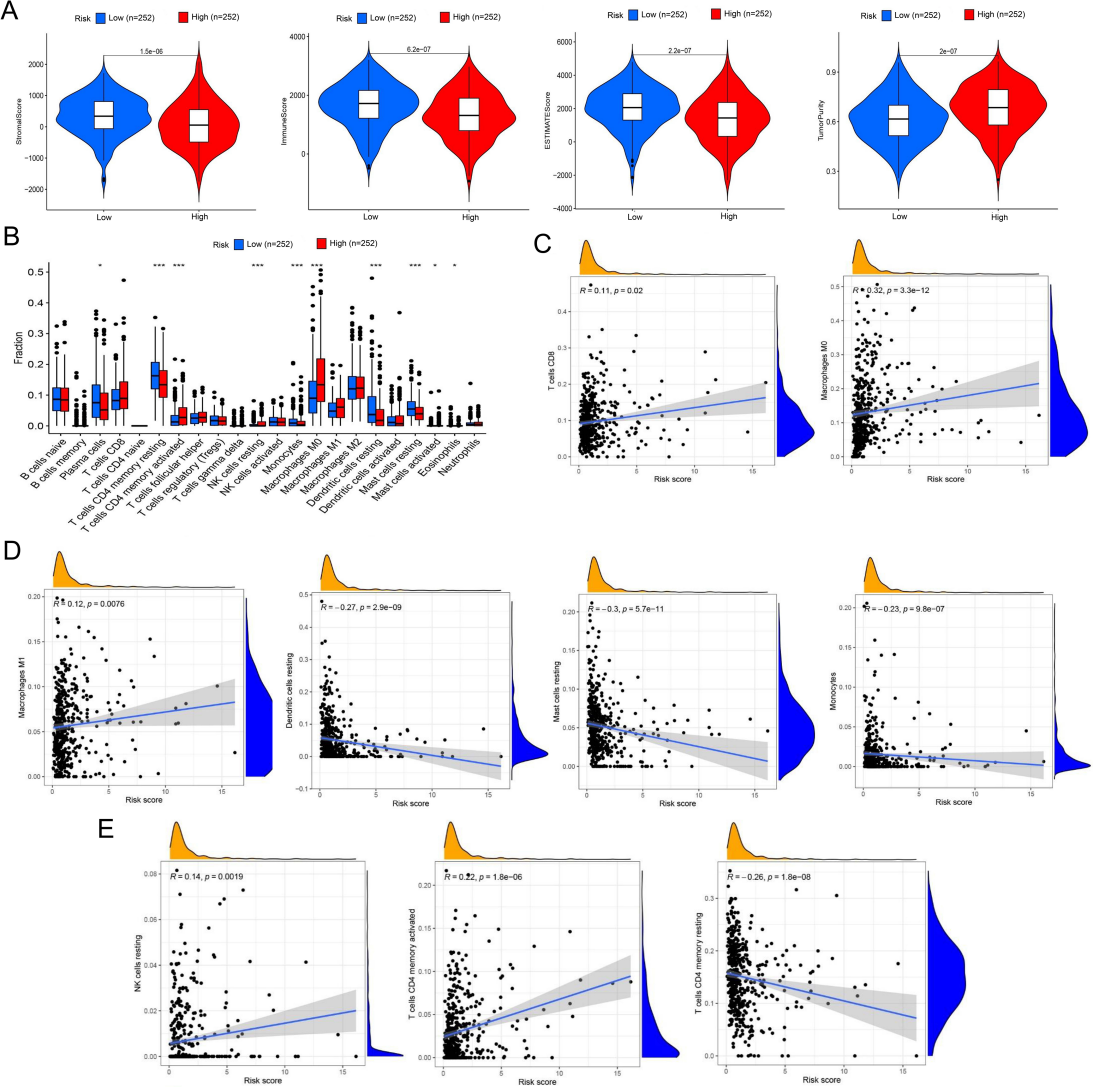
Comprehensive characterization of tumor microenvironment and immune cell infiltration stratified by risk groups in LUAD. **(A)** Violin plots quantitatively compare stromal score (reflecting extracellular matrix components), immune score (representing overall immune cell infiltration), tumor purity (estimated proportion of malignant cells), and ESTIMATE score (combined microenvironment evaluation) between high- and low-risk LUAD patients. **(B)** Boxplots with notches (representing 95% confidence intervals of the median) demonstrate statistically significant variations in 22 immune cell subtypes quantified by CIBERSORTx deconvolution algorithm between risk groups. **(C)** Scatter plots with locally weighted smoothing (LOESS) regression illustrate the non-linear associations between continuous risk scores and immune cell infiltration, revealing a positive correlation with CD8+ cytotoxic T cell abundance and a positive relationship with M0 macrophage infiltration. **(D, E)** Systematic immune correlation profiling. Expanded correlation matrices highlight risk-associated immune features. *P< 0.05 and *** *P<* 0.001.

We also validated the efficacy of the risk model in immune cell landscape characterization. High-risk patients (n = 252) showed lower stromal, immune, and summed scores and greater tumor purity (**Figure 4A**) than low-risk patients. High-risk patients displayed increased infiltration of memory activated CD4^+^ T cells, resting NK cells, M0 macrophages, and activated mast cells and inhibited infiltration of plasma cells, memory resting CD4^+^ T cells, monocytes, resting dendritic cells, resting mast cells, and eosinophils (**Figure 4B**). The correlation analysis of riskScore and immune cell infiltration showed that CD8+ T cell, M0/M1 macrophage, resting NK cell, and activated memory CD4+ T cell infiltration were enhanced, and resting memory CD4^+^ T cell, resting dendritic cell, resting mast cell, and monocyte infiltration pronouncedly decreased with increasing riskScore (**Figures 4C–E**).

**Figure 5:**
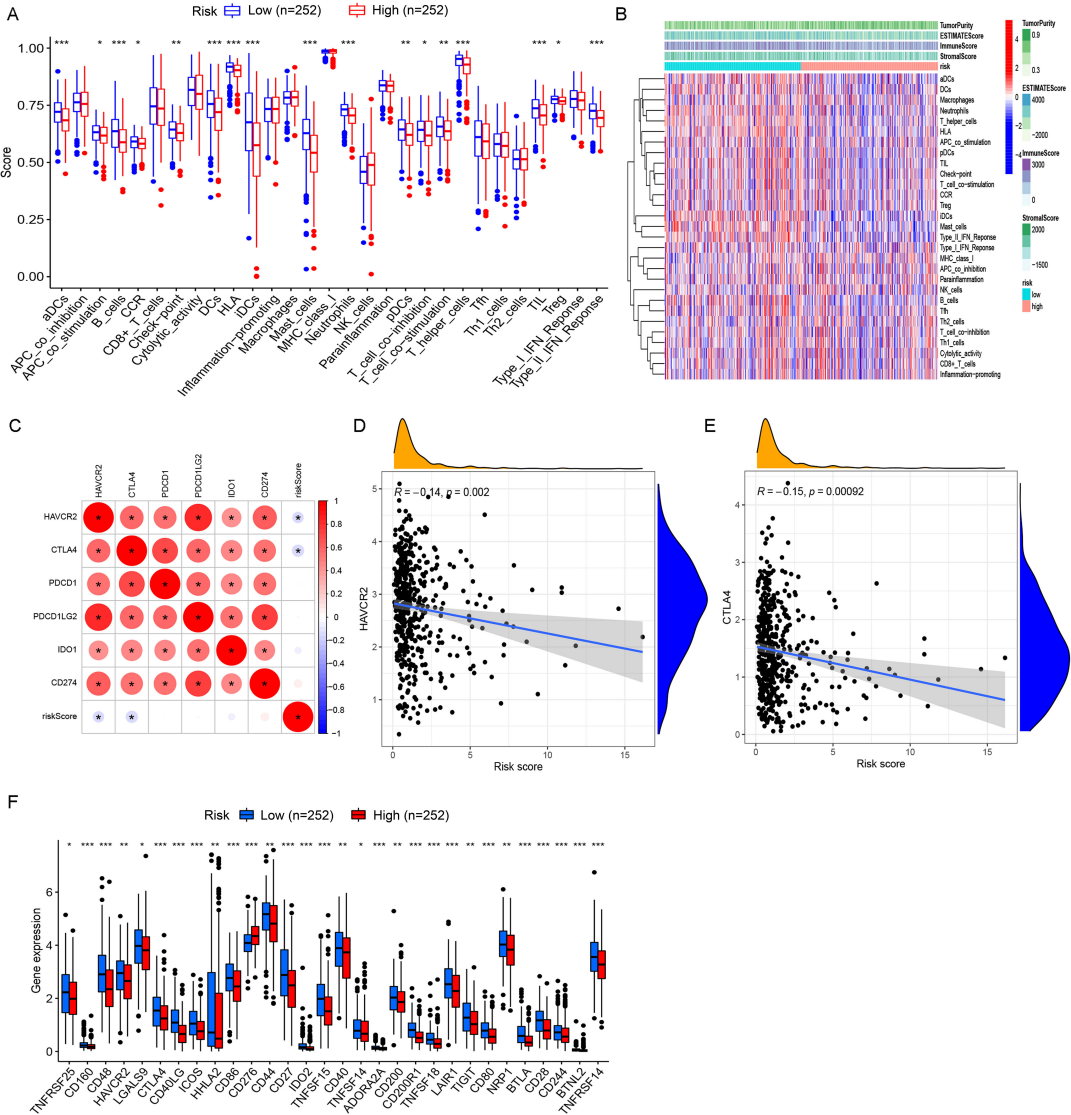
Comprehensive immune profiling and checkpoint expression patterns in LUAD risk groups. **(A)** Boxplots with overlaid individual data points compare the abundance of tumor-infiltrating immune cells and immune functional scores between high- and low-risk LUAD patients. **(B)** Multi-dimensional immune landscape characterization. The heatmap demonstrates systematic variations in immune signatures across risk groups. Columns represent patients (stratified by risk), whereas rows show immune features. **(C)** Spearman correlation matrix identifies clinically relevant immune checkpoints significantly associated with risk scores. Circle size represents correlation strength, while color indicates direction (red, positive; and blue, negative). **(D, E)** Key checkpoint expression dynamics. LOESS regression plots depict non-linear relationships between: **(D)***HAVCR2* expression and risk scores, with marginal histogram showing risk score distribution; **(E)***CTLA4* expression and risk scores. Both checkpoints show monotonic increases with risk progression. **(F)** Comparative boxplots confirm elevated expression of one clinically targetable checkpoints (CD276) in high-risk patients. *P< 0.05, **P< 0.01, and *** *P<* 0.001.

In ssGSEA analysis, immune scores of immune cells or their functions, including activated dendritic cells (aDCs), APC_co_stimulation, B cells, C-C chemokine receptor (CCR), Check-point, dendritic cells (DCs), human leukocyte antigen (HLA), immature dendritic cells (iDCs), Master-cell, Neutrophils, plasmacytoid dendritic cells (pDCs), T-cell_co_differentiation, T-cell_co_stimulation, T-helper-cells, tumor infiltrating lymphocytes (TIL), regulatory T cell (Treg), and Type-II–IFN-Repon, were significantly higher in high- vs. low-risk patients (**Figure 5A**). The immune scoring thermogram also revealed a decreased number of most types of immune cells alongside inhibited immune cell functions in the high-risk group (**Figure 5B**). The analysis identified 30 differentially expressed immune checkpoint genes, comprising 29 downregulated genes (*TNFRSF25*, *CD160*, *CD48*, *HAVCR2*, *LGALS9*, *CTLA4*, *CD40LG*, *ICOS*, *HHLA2*, *CD86*, *CD44*, *CD27*, *IDO2*, *TNFSF15*, *CD40*, *TNFSF14*, *ADORA2A*, *CD200*, *CD200R1*, *TNFSF18*, *LAIR1*, *TIGIT*, *CD80*, *NRP1*, *BTLA*, *CD28*, *CD244*, *BTNL2*, and *TNFRSF14*) and one upregulated gene (*CD276*) in the high-risk group (**Figures 5C–E**). Notably, *HAVCR2* and *CTLA4* expressions showed progressive downregulation with increasing riskScore (**Figure 5F**).

### 3.4 Prognostic single genes in LUAD

**Figure 6:**
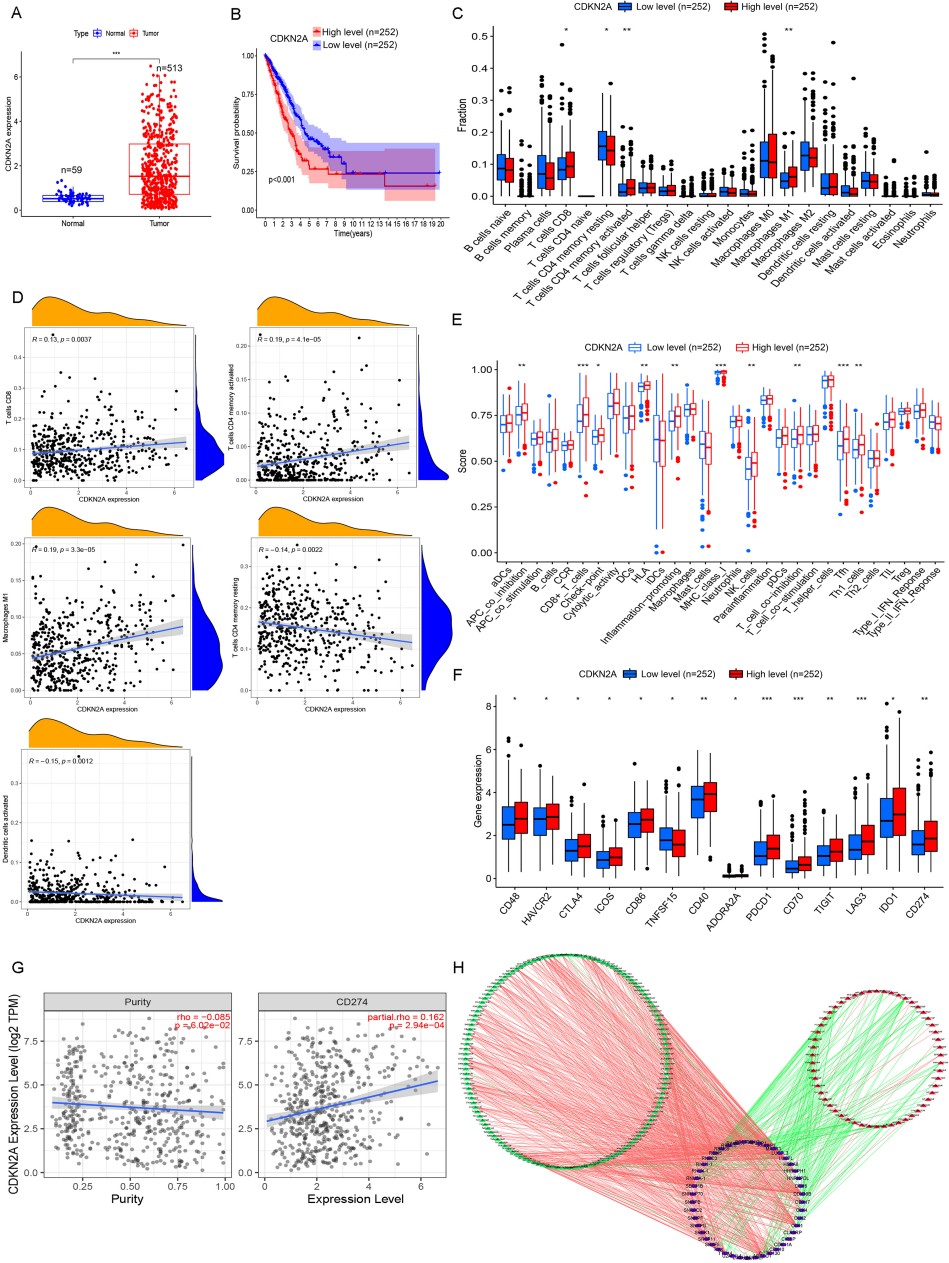
Comprehensive analysis of *CDKN2A* in high- and low-risk LUAD patients. **(A)** Differentially expressed genes (DEGs) between high- and low-risk LUAD groups reveal key transcriptional alterations linked to *CDKN2A* status. **(B)** Kaplan–Meier survival analysis demonstrates distinct clinical outcomes between tumor tissues and adjacent normal tissues when stratified by *CDKN2A* expression levels. **(C)** Comparative analysis of immune cell infiltration patterns shows significantly different immune microenvironment compositions between *CDKN2A* high- and low-expression groups. **(D)** Correlation analysis elucidates the quantitative relationship between *CDKN2A* expression levels and immune cell abundance in the tumor microenvironment. **(E)** Comprehensive evaluation of immune cell subpopulations and functional activity scores demonstrates *CDKN2A*-dependent immunomodulatory effects. **(F)** Systematic comparison of immune checkpoint molecule expression (PD-1, CTLA-4, etc.) reveals differential immune evasion potential between *CDKN2A* expression groups. **(G)** Scatter plot analysis identifies a significant correlation between *CDKN2A* expression and *CD274* (PD-L1) levels, suggesting potential implications for immunotherapy response. **(H)** The SF-AS regulatory network analysis identifies 188 prognosis-associated splicing events (54 high-risk and 134 low-risk) coordinated by 45 splicing factors, providing mechanistic insights into *CDKN2A*-mediated LUAD pathogenesis through alternative splicing regulation. *P< 0.05, **P< 0.01, and ***P< 0.001.

Among PASE-related genes, cyclin-dependent kinase inhibitor 2A (*CDKN2A*) showed the most significant upregulation in tumor tissues compared with adjacent normal tissues (|logFC| = 1.38, *P*< 0.001) (**Figure 6A**). Therefore, all LUAD patients included were assigned to the high (n = 252)– or low (n = 252)–expression group according to the median expression score of *CDKN2A*. Kaplan–Meier survival curves showed that patients with *CDKN2A* overexpression had worse OS (**Figure 6B**), indicating the prognostic significance of the *CDKN2A* gene in LUAD.

Then, the correlation of *CDKN2A* expression with immune cell infiltration was assessed. High *CDKN2A* expression was significantly associated with increased CD8^+^ T cell, activated memory CD4^+^ T cell and M1 macrophage infiltration, and suppressed resting memory CD4^+^ T cell and dendritic cell infiltration in LUAD (**Figures 6C, D**). Patients with high *CDKN2A* expression showed higher immune scores of APC_co_differentiation, CD8^+^-T-cells, Check-point, HLA, Infection-promotion, MHC-class-I, NK-cells, T-cell_co_differentiation, Tfh, and Th1-cells compared to those with low *CDKN2A* expression (**Figure 6E**). Fourteen immune checkpoint genes were found differentially expressed in LUAD, comprising 13 upregulated genes (*CD48*, *HAVCR2*, *CTLA4*, *ICOS*, *CD86*, *CD40*, *ADORA2A*, *PDCD1*, *CD70*, *TIGIT*, *LAG3*, *IDO1*, and *CD274*) and the downregulated *TNFSF15* in the high expression group (**Figure 6F**). The co-expression of *CDKN2A* and the immune checkpoint gene *CD274* was enhanced with elevated tumor purity (**Figure 6G**).

The SF-AS regulatory network was characterized to provide a glimpse into the potential mechanisms of LUAD tumorigenesis. We identified 188 PASEs, including 54 high-risk events and 134 low-risk events, which were associated with 45 SFs in LUAD by correlation analysis between SFs and PASEs. A total of 135 PASEs were positively correlated with 43 SFs, and 53 PASEs were negatively correlated with 38 SFs (**Figure 6H**). Overall, most high-risk PASEs were associated with inhibited expressions of SFs (**Figure 6H**). Among these SFs, family members such as *SRSF5* and *SRSF11* showed positive correlations with multiple high-risk PASEs, suggesting that these factors may promote the occurrence of oncogenic splicing variants. Notably, *SRSF11* demonstrated strong positive correlations with aberrant splicing events of genes including *METTL3* (R = 0.65) and *METTL17* (R = 0.65), which may represent one of the key mechanisms underlying the dysregulated expression of multiple PASEs in LUAD. The correlation analysis results between SFs and PASEs are presented in **Supplementary Table 1**.

### 3.5 Validation study

**Figure 7:**
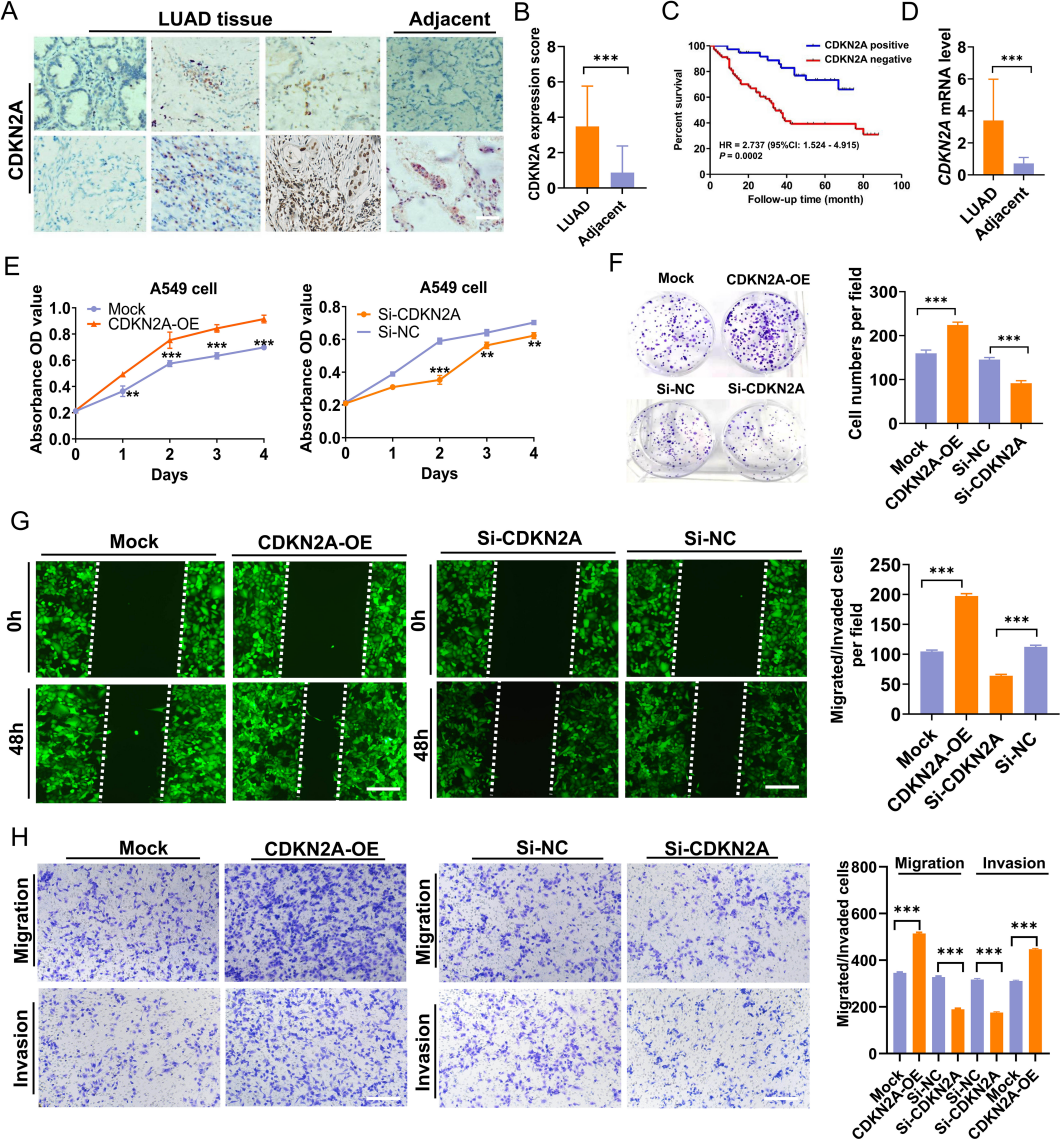
Elevation of CDKN2A expression in LUAD and its biological function in A549 cells. **(A, B)** CDKN2A expression levels were compared between LUAD and adjacent noncancer tissues [individual cohort form a tissue chip (Cat. No. HLugA180Su05), comprising 94 LUAD and 86 adjacent tissue spots; Shanghai Outdo Biotech, Shanghai, China]. **(C)** An inverse correlation was observed between increased CDKN2A expression and overall survival (OS) time. **(D)** Elevated *CDKN2A* mRNA levels in LUAD were validated using a cDNA chip (Cat. No. cDNA-HLugA030PG01, Shanghai Outdo Biotech, Shanghai, China; each tissue chip has 15 LUAD tissue points together with 15 adjacent normal tissue points). **(E)** CCK-8 assays were conducted to assess the viability of A549 cells with CDKN2A overexpression (CDKN2A-OE) or CDKN2A knockdown (Si-CDKN2A). **(F)** Colony formation ability was evaluated. **(G, H)** Migration and invasion capacities of A549 cells were assessed through Transwell and wound healing assays, employing stable transfections with CDKN2A-OE, Mock, Si-CDKN2A, and negative control siRNA (Si-NC) vectors. All data are presented as means ± SDs. Statistical analysis was performed using Student’s t-test or the Mann – Whitney test in **(B, D–H)**; Kaplan–Meier analysis was used in **(C)**. **P< 0.01, and ***P< 0.001.

Validation was performed using LUAD tissue and cDNA microarrays. Immunohistochemistry (IHC) and subsequent analyses revealed that CDKN2A protein predominantly localized in the cytoplasm of LUAD cells (**Figure 7A**). Of the 94 LUAD cases examined, 22 exhibited high CDKN2A expression, and 57 displayed low to medium expression, including fifteen CDKN2A-negative cases. The positive expression rate of CDKN2A in LUAD was 84.04% (79/94), significantly exceeding the 47.67% (41/86) observed in adjacent non-tumor tissues. Moreover, the average expression level (rating score) of CDKN2A were elevated in LUAD tissues compared to those in adjacent non-tumor tissues (**Figure 7B**). Survival analysis indicated that patients with high CDKN2A expression tended to have worse prognoses and shortened OS time (hazard ratio = 2.737; 95% confidence interval: 1.524–4.915; *P* = 0.0002) (**Figure 7C**). Prognostic analysis using Cox proportional hazards regression models to evaluate the association between clinicopathological variables and survival outcomes in LUAD patients is shown in **Table 1**. Correspondingly, qRT-PCR analysis indicated that *CDKN2A* expression was significantly higher in LUAD tissues than in matched control samples (**Figure 7D**).

**Table 1:** Cox regression analysis of clinicopathological variables and survival in LUAD.

Clinical parameters	HR	95%CI	*P* value
Sex (Male vs. Female)	0.772	0.445-1.340	0.3580
Age (>60 years vs. ≤60 years)	1.009	0.605-1.683	0.9740
Clinical stage (I+II *vs.* III+IV)	2.338	1.148-4.763	0.0190
T stage	0.930	0.451-1.917	0.8440
N stage	2.199	1.213-3.990	0.0090
EGFR mutation (Positive vs. Negative)	1.199	0.615-2.338	0.5940
CDKN2A (High vs. Low)	2.737	1.524-4.915	0.0002

LUAD, lung adenocarcinoma; HR, hazard ratio; CI, confidence interval; EGFR, epidermal growth factor receptor; CDKN2A, cyclin-dependent kinase inhibitor 2A.

To determine whether CDKN2A could influence the LUAD cell phenotype, we conducted both overexpression and knockdown experiments in A549 LUAD cells, which yielded relatively moderate CDKN2A expression. Results indicated that overexpression of CDKN2A promoted growth, proliferation, clonogenicity, as well as invasion and migration capabilities of A549 cells; conversely, knocking down CDKN2A expression in A549 cells reduced their growth, proliferation, clonogenic formation, and their *in vitro* invasion and migration capabilities (**Figures 7E–H**). Collectively, these results suggest that CDKN2A acts as a tumor promoter that facilitates LAUD progression *in vitro.*

### 3.6 Prediction of targeted drugs of CDKN2A and the antitumor effects *in vivo*

**Figure 8:**
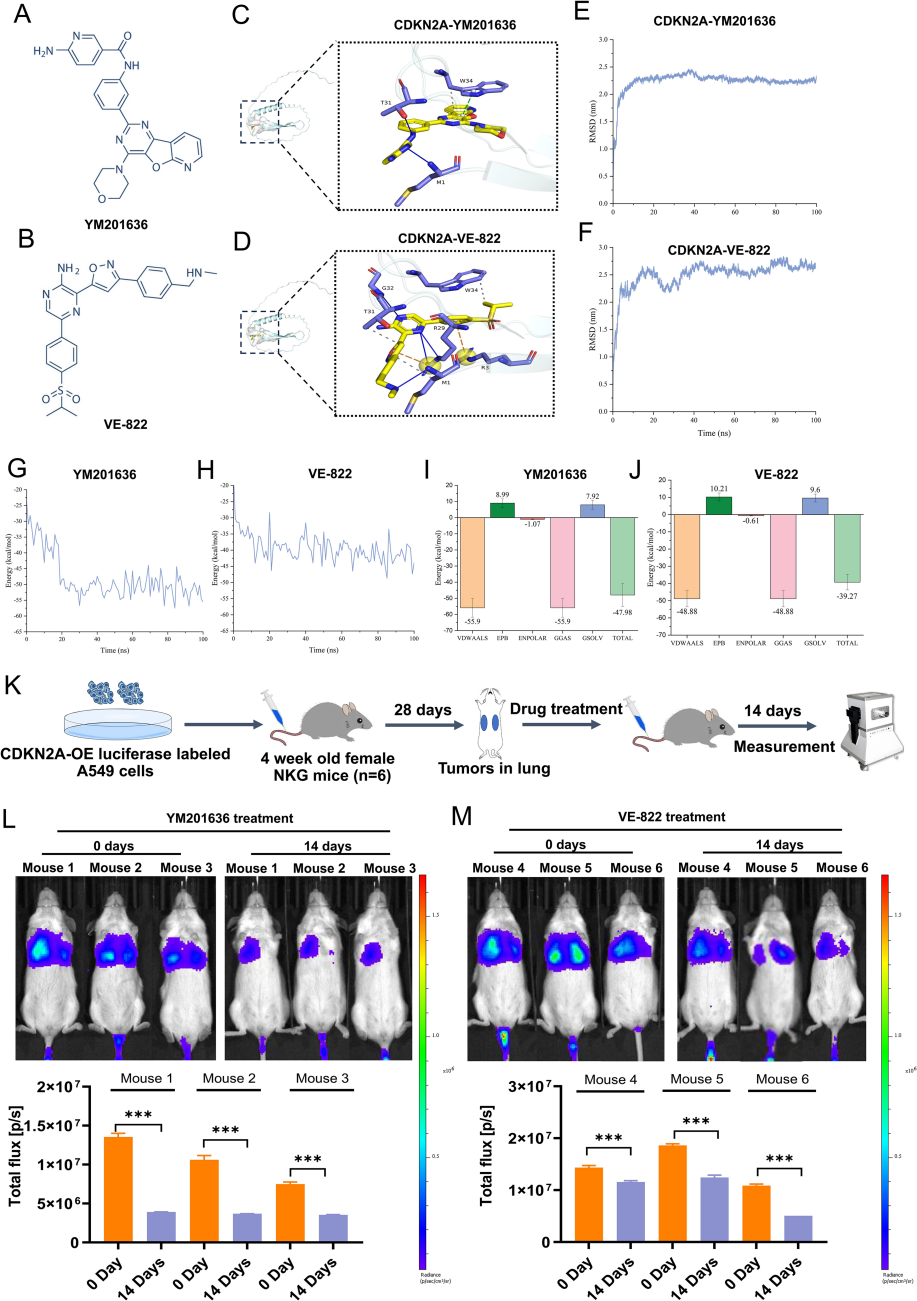
Screening of targeted drugs for CDKN2A through molecular docking and kinetic analysis, and their antitumor effects *in vivo*. **(A, B)** Structural formulas of the identified drugs YM-201636 and VE-822 are shown. **(C, D)** 3D diagrams depict the binding of YM-201636 and VE-822 to CDKN2A. **(E, F)** RMSD behavior of YM-201636 and VE-822 in molecular dynamics simulations is displayed. **(G, H)** The single-trajectory method was used to calculate the interaction energy of the protein–small-molecule drug complex. **(I, J)** The energy contributions of van der Waals (VDWAALS), electrostatic energy (EPB), polarization energy (ENPOLAR), gas phase energy (GGAS), and solvation energy (GSOLV) were calculated. **(K)** A schematic diagram illustrating the antitumor effects *in vivo* is presented. **(L, M)** Alterations in tumor fluorescence signal before and after treatment with YM-201636 or VE-822 are shown. All results are expressed as means ± SDs. Statistical analysis was performed using Student’s t-test or the Mann – Whitney test in **(L, M)**. ***P< 0.001.

Molecular docking was utilized to forecast the potential therapeutic efficacy and plausible mechanism of CDKN2A in treating LUAD. Among the findings for CDKN2A wild-type and prospective small-molecule inhibitors, YM-201636 and VE-822 (Berzosertib) emerged as the top two small molecules (**Figures 8A, B**). Additionally, a 3D representation of YM-201636 and VE-822 binding to CDKN2A is depicted in **Figures 8C, D**, respectively. The screening process and results of the candidate small-molecule inhibitors of CDKN2A protein is shown in **Supplementary Figure 1**. Additional candidate small-molecule inhibitors that were screened are listed in **Supplementary Table 1**.

MDS analysis was conducted to corroborate the binding capabilities between CDKN2A and the top two drugs (YM-201636/VE-822). The drug discovery workflow (including primary screening data) for CDKN2A-targeting compounds is detailed in **Supplementary Figure 1** and **Supplementary Table 2**. The RMSD behavior of the two complexes, CDKN2A-YM201636 and CDKN2A-VE-822, exhibited similarity: initially, RMSD values surged rapidly, signifying the system’s adaptation toward a more stable configuration (**Figures 8E, F**). Subsequently, the equilibrium stage was reached, where the RMSD value stabilized, indicating that the atomic interactions had attained an equilibrium state. The CDKN2A-YM201636 complex demonstrated an average interaction energy of −47.98 kcal/mol, whereas the CDKN2A-VE-822 complex exhibited an average interaction energy of −39.27 kcal/mol, and both complexes displayed a relatively stable binding state (**Figures 8G, H**). The results indicated that van der Waals forces predominantly contributed to the interaction energy. Overall, these findings suggest that both CDKN2A-YM201636 and CDKN2A-VE-822 possess favorable binding abilities (**Figures 8I, J**).

To further assess the antitumor activity of YM201636 and VE-822, additional orthotopic mouse models of LUAD were established using CDKN2A-OE luciferase-labeled human A549 cells delivered by tail vein injection (**Figure 8K**). After treatment with YM201636 (2 mg/kg) or VE-822 (25 mg/kg) via tail vein injection, the fluorescence intensity of LUAD tumors treated with both drugs was significantly reduced (**Figures 8L, M**). These findings indicate that YM201636 and VE-822 may serve as potential CDKN2A-targeted therapies for LUAD treatment.

## 4 Discussion

Currently, the majority of patients with LUAD subtypes that present with metastatic disease cannot yield a satisfactory response to anticancer therapies. A new prognostic marker for risk prediction of OS and TIME characterization to identify high-risk patients early on and timely monitor individualized treatment is necessary. Notably, complex mechanisms controlling LUAD tumorigenesis and progression (27), such as AS events producing multiple mRNA isoforms and protein diversity, means that a network-based signature may be more capable of predicting patient survival accurately and specifically than a multigene signature.

However, most studies focusing on gene mutation and transcription fail to explain why the same mutated gene may induce various or even opposite carcinogenic effects in different tumor types or problems alike (28, 29). AS events can be a plausible answer to the question. Their roles in the occurrence and prognosis of LUAD have been previously proven (30, 31). In the present study, we explored PASEs related to LUAD and identified 13 key events (BEST3|23,330|AT, CDKN2A|86,004|AP, PKIB|77,377|AP, TTC39C|44,852|AP, MEGF6|315|ES, HNRNPLL|53,258|AT, CA5B|98,313|ES, LDB1|12,935|AP, C12orf76|24,406|AT, AP2B1|40,327|AD, LETM2|83,398|AT, MKL1|62,348|AP, and RPL33|53,046|ES) for risk model establishment. The 513 LUAD patients were stratified into high- and low-risk groups based on the median riskScore cutoff. Kaplan–Meier analysis confirmed significantly poorer OS in high-risk patients, with mortality rising alongside riskScore. Univariate and multivariate Cox regression demonstrated the risk model’s independent prognostic value, outperforming clinical factors in predictive accuracy (AUCs > 0.75). Notably, riskScore correlated with advanced tumor stage, male gender, and lymph node metastasis, reinforcing its clinical relevance. The nomogram integrating these factors exhibited robust predictive performance, validated by calibration curves. These findings underscore the model’s utility in stratifying LUAD prognosis.

LUAD patients often have a varying response to immunotherapy, which primarily depends on their immune cell landscape (32). Infiltrated immune cells in some malignant tumors have been proven to significantly impair immunotherapy response and patient prognosis (33–35). In the TIME, the immune cell landscape is a critical link for clinical outcomes as infiltrated immune cells may improve or impair the immune response and angiogenesis directly or indirectly (36). Numerous studies have shown that tumor infiltration of CD8+ T cells is significantly associated with the prognosis of lung carcinoma patients (37). Circulating macrophages infiltrate tumor tissues and differentiate and mature into tumor-associated macrophages triggered by inhibitory cytokines derived from tumor cells and the TIME to promote tumor angiogenesis and lymphangiogenesis (38). In this study, we assessed the relationship between the riskScore and immune cell infiltration and found a milieu favorable for tumor immune escape, like the increased infiltration of CD8^+^ T cells, M0/M1 macrophages, resting NK cells, and activated memory CD4^+^ T cells and inhibited resting memory CD4^+^ T cell, resting dendritic cell, resting mast cell, and monocyte infiltration with increasing riskScore. Our ssGSEA results also revealed critical differences in immune landscapes between risk groups. The low-risk group demonstrates significantly enhanced immune activity across multiple cell types (aDCs and B cells) and functional pathways (antigen-presenting cell (APC) co-stimulation, type-II IFN response), whereas high-risk patients exhibit broad immunosuppression with coordinated downregulation of effector cells and immunostimulatory pathways. Notably, we observed differential expression of 30 immune checkpoint genes, with 29 (including CTLA4 and HAVCR2) progressively downregulated and only CD276 upregulated in high-risk patients. These patterns suggest that 1) our riskScore quantitatively reflects both functional immune impairment and regulatory alterations; 2) the high-risk microenvironment exhibits global immune dysfunction beyond individual checkpoint changes; and 3) CD276 upregulation may represent a compensatory mechanism. These findings have important implications for immunotherapy stratification, particularly the observed progressive immune exhaustion with increasing riskScore. The coordinated downregulation of multiple inhibitory checkpoints (CTLA4, HAVCR2, and TIGIT) in high-risk patients may explain their poorer prognosis while suggesting potential resistance mechanisms to current immunotherapies. These findings suggest the prognostic effect of immune cell infiltration in tumors. The prognostic model we established can serve as a tool to offer a window into the mechanisms for the pro-tumorigenic immune microenvironment.

Genes related to the 13 PASEs included in the risk model have been shown to have close associations with tumor occurrence and progression. Among them, only *CDKN2A* was differentially expressed in LUAD. The *CDKN2A* gene, or multiple tumor suppressor 1 (*MTS1*), is an 8,500-bp gene located at 9p21 in humans (39), contains three exons, and encodes p16INK4a protein (40). p16INK4a is a cell cycle–dependent protein as it binds to CDK4 and CDK6 and inhibits the kinase activity of cyclin/CDK4 complexes to block Rb phosphorylation and induce cell-cycle arrest at G phase, thus inhibiting cell proliferation (41). Mutations in the *CDKN2A* gene often lead to loss of anti-cancer activity of *CDKN2A*, thus promoting cell proliferation and tumorigenesis (42). Tumor cells often show faster proliferation and differentiation than normal cells, which may be, in part, explained by *CDKN2A* mutations or inactivation (43). In LUAD, some study has shown that the frequency of *CDKN2A* mutations is higher in lung carcinoma tissues than in tumor-adjacent normal tissues, indicating that *CDKN2A* mutations can be associated with tumorigenesis (44). Thus, the *CDKN2A* gene is expected to be a new direction of molecular targeted therapy for LUAD.

In this study, we also found that *CDKN2A* was positively correlated with the expression of an important immune checkpoint gene *CD274*. Currently, only a few studies reported the expression pattern of *CDKN2A* in LUAD (45), and the relationship between CDKN2A and the development and prognosis of LUAD as well as the functional role is uncertain. Our analysis showed that *CDKN2A* gene upregulation was closely associated with reductions in immune effector cells and their functions, aberrant immune checkpoint gene expressions, and worse OS of patients. We further validated the expression and prognostic significance of CDKN2A in LUAD through reverse transcription quantitative polymerase chain reaction (RT-qPCR) and IHC. The results indicated an increase in CDKN2A in LUAD tissues; correspondingly, the CDKN2A protein was also upregulated in LUAD tissue, and patients exhibiting higher CDKN2A expression exhibited shortened OS time. Additionally, a pan-cancer study has demonstrated that the total expression of CDKN2A was significantly elevated in LUAD (46). In SCLC, it has been reported that *CDKN2A* mRNA levels in 357 SCLC cases were notably higher than those in the control group, and patients with higher CDKN2A expression had considerably poorer OS rates compared to those with lower CDKN2A levels (47). Our *in vitro* study further revealed that CDKN2A displayed oncogenic biological behavior in LUAD. Contrary to our *in vitro* results, Liu et al. reported that *CDKN2A* silencing stimulates cell proliferation, migration, and invasion in both A549 and H322 cells (48). We consider that factors such as differing cell sources and knockdown sequences may account for these disparate effects. Notably, our study pioneeringly confirmed that CDKN2A can serve as a drug target in the treatment of LUAD. Furthermore, YM201636 and VE-822, both high-affinity drugs that bind to CDKN2A, exhibited promising anti-tumor effects *in vivo*. Our study reveals distinct antitumor mechanisms of YM201636 and VE-822 in lung cancer. YM201636, a PIKfyve inhibitor, demonstrates dose-dependent suppression of NSCLC cell proliferation with cell-type–specific claudin modulation (upregulating CLDN1/3/5 in HCC827, CLDN3/5 in Calu-1, and CLDN5 with CLDN1 reduction in H1299) and consistent *epidermal growth factor receptor* (*EGFR*) mRNA induction, suggesting PIKfyve-EGFR crosstalk in EGFR-activated NSCLC (49). Meanwhile, the ataxia telangiectasia and Rad3-related protein (ATR) inhibitor VE-822 exhibits LUAD suppression through a novel OTUD1-FHL1 axis, inhibiting tumor growth both *in vitro* and *in vivo* (50). While our preliminary data suggest CDKN2A involvement, the cell-specific claudin effects and VE-822’s dual mechanism as both ATR inhibitor and OTUD1 activator warrant further investigation in clinically relevant models to assess their therapeutic potential, particularly in CDKN2A-altered tumors and combination regimens.

This study has several important limitations that warrant consideration. The experimental scope was constrained by a relatively small tissue sample size and the use of only one cell line for functional validation, which may limit the generalizability of our findings. Additionally, the *in vivo* experiments involved a restricted number of animal subjects, potentially affecting the statistical power of those results. While our risk scoring system shows promising correlations with immune microenvironment features and demonstrates capacity for immunological stratification, these observations currently rely exclusively on computational analyses and preclinical data, lacking validation in clinical immunotherapy cohorts. Although we have begun assembling a cohort of anti-PD-1-treated patients and designed follow-up validation protocols, the model’s true predictive value for immunotherapy responses cannot be definitively established until rigorous clinical correlation studies are completed with actual treatment outcome data. Lastly, the binding affinity and specificity between YM-201636/VE-822 and CDKN2A protein require further validation through surface plasmon resonance, isothermal titration calorimetry, or co-crystallization experiments. These methodological constraints highlight the need for more comprehensive, large-scale investigations to both verify our current findings and further elucidate the multifaceted role of CDKN2A in LUAD pathogenesis and treatment response. Future studies incorporating diverse cell models, expanded clinical samples, and prospective validation cohorts will be essential to translate these preliminary findings into clinically applicable biomarkers.

In conclusion, PASEs offer novel insights into the TIME during the tumorigenesis and progression of LUAD. The PASE-based risk model can forecast prognosis, can delineate the immune cell signature of LUAD patients, and can emerge as a promising biomarker for personalized immunotherapy monitoring in clinical settings. Furthermore, genes associated with PASEs represent potential oncogenic drivers that merit further investigation. Notably, CDKN2A functions as an oncogene and could be considered a promising drug target for LUAD.

## Data Availability

The datasets presented in this study can be found in online repositories. The names of the repository/repositories and accession number(s) can be found in the article/**Supplementary Material**.
